# MicroRNA-125b-5p regulates IL-1β induced inflammatory genes via targeting TRAF6-mediated MAPKs and NF-κB signaling in human osteoarthritic chondrocytes

**DOI:** 10.1038/s41598-019-42601-3

**Published:** 2019-05-03

**Authors:** Zafar Rasheed, Naila Rasheed, Waleed Al Abdulmonem, Muhammad Ismail Khan

**Affiliations:** 1 Department of Medical Biochemistry, College of Medicine, Buraidah, Qassim University, Buraidah, Saudi Arabia; 2 Department of Pathology, College of Medicine, Buraidah, Qassim University, Buraidah, Saudi Arabia; 3 Faculty of Medicine, School of Public Health, University of Queensland, Brisbane, Australia

**Keywords:** Non-coding RNAs, RNAi

## Abstract

Abnormal post-transcriptional modulations in inflammatory genes by microRNAs (miRNAs) play a crucial role in human disorders including arthritis. In this study, we determined the effect of hsa-miR-125b-5p on interleukin (IL)-1β induced inflammatory genes in human osteoarthritic (OA) chondrocytes. Bioinformatics algorithms showed 3′untranslated region (3′UTR) of TRAF6 mRNA (NM_004620.3) has perfectly matched ‘seed-sequence’ for hsa-miR-125b-5p. Treatment of cells with IL-1β up-regulates TRAF6 mRNA and down-regulates hsa-miR-125b-5p expression. This negative correlation between TRAF6 and hsa-miR-125b-5p was verified by transfection with miR-125b mimic (pre-miR-125b). Moreover, transfection with miR-125b mimic caused marked inhibition of IL-1β-induced phosphorylation of p38-MAPK, JNK-MAPKs and ERK-MAPKs and also suppressed the nuclear levels of NF-κBp50, NF-κBp65 and inhibited the activation of IκBα. Furthermore, transfected chondrocytes with miR-125b mimic in the presence of IL-1β also showed marked inhibition in the secretion of several proinflammatory cytokines, chemokines and growth factors including IL-6, IL-8, INF-γ, TGF-β1, IGFBP-1 and PGDF-BB. Importantly, this transfection also significantly inhibited IL-1β- induced MMP-13 expression/production. In short, this study concludes that hsa-miR-125b-5p acts as a negative co-regulator of inflammatory genes including MMP-13 via targeting TRAF6/MAPKs/NF-κB pathway in human OA chondrocytes.

## Introduction

Osteoarthritis (OA) is the most common health problems of the joints, affecting individuals globally. Its onset occurs when the breakdown of the cartilage tissue begins. Although in OA, any joint tissues can be damaged, but it generally effects on the knees and the hips^1,2^. It is now well established that the mechanisms occur in OA are multifactorial, but its etiology remains to be fully explored^1,2^. The cartilage in the joints is mainly comprised of a condensed extracellular matrix (ECM) with a random scattering of highly specialized cells known as articular chondrocytes^3^. Articular chondrocytes are well known single cell type of the cartilage that maintain its hemostasis by the regeneration of the constituents of ECM and the cartilage degrading enzymes^3^ and now this cell type becomes the first choice to study and to understand the pathogenesis involved in OA. The molecular evidences indicate that the pathogenesis of OA is well linked with the overproduction of potent inflammatory cytokine IL-1β, which plays an important function in the cartilage breakdown through upregulation of potent cartilage degrading enzymes including aggrecanases, matrix metalloproteinase (MMP)s and also promotes productions of other mediators of inflammation including proinflammatory cytokines, chemokines and several growth factors known to involve in cartilage degeneration^2,4–6^.

MicroRNAs (miRNA) are non-coding small nucleic acids play important role in modulation of their target genes by binding with their complementary sequences at 3′untranslated regions (3′UTR) during the post transcriptional processing^7^. In the recent years, several miRNAs were defined and now it is expected that about more than 30% of all mammalian genes are regulated by miRNAs^8^. So far, the function of miRNAs was discovered in several human disorders and several miRNAs were already reported to regulate the disease modifying genes^7,8^ and now we believe that the miRNA regulation is not only important for the disease detection but also for the therapeutic applications.

In OA, the regulatory function of miRNAs has somewhat defined in the cartilage pathophysiology^9,10^. Studies showed the involvement of miRNAs in several stages of cartilage development, homeostasis, and disease onset^10,11^. In our earlier studies, we characterized the global expression of miRNAs in stimulated human OA chondrocytes^12^. In another study, we showed that the expression of an enzyme inducible nitric oxide synthase (iNOS) is regulated by hsa-miR-26a-5p through direct recognition with its 3′-UTR in human OA chondrocytes^13^. Moreover, we also demonstrated in human OA chondrocytes that inflammatory cell signaling is linked with the negative co-relation of hsa-miR-26a-5p and iNOS^13^. Furthermore, we also demonstrated that miRNAs hsa-miR-199a-3p and hsa-miR-140-3p negatively regulate COX-2 and ADAMTS-5 expression, respectively in human OA chondrocytes^14,15^. Recently, the function of miR-125b was discovered in various cell types and its association with several human disorders was reported^16–19^. In OA, the function of miR-125b was somewhat defined by few studies only, in one study miR-125b was reported to regulate aggrecanase-1 expression in human chondrocytes^20^ and in another study, miR-125b was reported to regulate the inflammatory activities in stimulated chondrogenic cells via MIP-1α signaling event^21^. These reports clearly pointing out the importance of miR-125b in OA, but need to be further investigated.

In arthritic joints, chondrocytes are well known to secrete number of proinfammatory mediators extensively including MMP-13 and an overproduction of MMP-13 in the joints are known to promote cartilage breakdown and to induce join pain^22^. Now we believe that strategy that target MMP-13 is a most powerful way to manage the onset of joints pain in OA. In one of our previous studies, we discovered that miRNA hsa-miR-27b-3p regulates MMP-13 expression/production via direct binding with its 3′UTR in human OA chondrocytes^23^. Now it is also known that the degeneration of arthritic joints is well linked to the abnormal production of proinflammatory cytokines, chemokines and various growth factors^5,24^ and in chondrocytes IL-1β is well-known to increase the secretion of several potent cytokines, chemokines and growth factors^5,6^. In view of the recently discovered role of hsa-miR-125b-5p in cartilage biology and the overproduction of MMP-13 or other mediators of inflammation in arthritic joints here we demonstrated that whether MMP-13 expression/production or production of proinflammatory cytokines, chemokines or growth factors have been regulated by hsa-miR-125b-5p in human OA chondrocytes stimulated with IL-1β. Our findings show that the hsa-miR-125b-5p regulates MMP-13 expression/production and several proinflammatory cytokines, chemokines or growth factors through TRAF6 mediated MAPKs and NF-κB signaling in stimulated human OA chondrocytes.

## Results

### Bioinformatics approach for the determination of hsa-miRNA-125b-5p target genes

**Figure 1:**
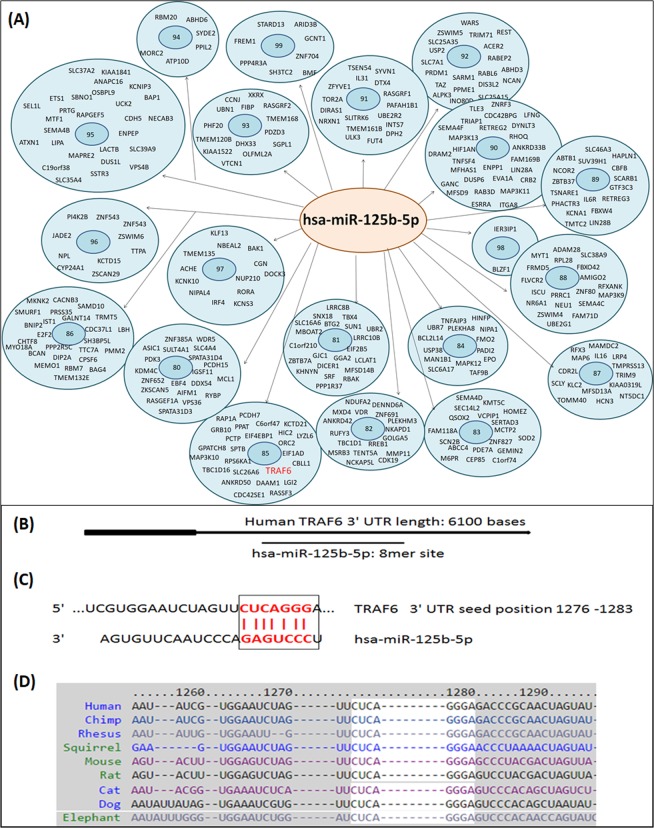
Expression of hsa-miR-125b-5p and TRAF6 in smooth and damaged OA cartilage tissues. (**A**) Smooth and damaged cartilage tissues from OA patients (**B**) Expression of hsa-mir-125b-5p in smooth and damaged cartilage tissues. ^#^p < 0.01 versus smooth cartilage tissue. (**C**) Gene Expression of TRAF6 in smooth and damaged cartilage tissues. ^@^p < 0.01 versus smooth cartilage tissue. (**D**) Protein expression of TRAF6 in smooth and damaged cartilage tissues (full length blot is provided in Supplementary Information File). ^*^p < 0.01 versus SC. SC, smooth cartilage; DC, damaged cartilage. Band images were digitally captured by the Un-Scan-It software and the band intensities of TRAF6 proteins were divided by band intensities of β-actin and are expressed in average pixels.

We used microRNA database (http://mirdb.org/) to determine hsa-miR-125b-5p predicted target genes. The microRNA database showed 925 target genes for hsa-miR-125b-5p, out of them top 335 genes with their target scores are summarized in Fig. 1A. Target score of TRAF6 gene for hsa-miR-125b-5p was found to be 85 (Fig. 1A). This microRNA database prediction of TRAF6 and hsa-miR-125b-5p was further confirmed by the TargetScan bioinformatics algorithm. The TargetScan approach was used to determine the binding probability between 3′UTR of TRAF6 mRNA (NM_004620.3) and hsa-miR-125b-5p. The data identified that the sequence conserved in the 3′UTR of TRAF6 mRNA was complementary with hsa-miR-125b-5p seed sequence. The complete TargetScan analysis is summarized in Fig. 1B–D.

### Levels of hsa-miR-125b-5p and TRAF6 in damaged and smooth cartilage of osteoarthritis patients

**Figure 2:**
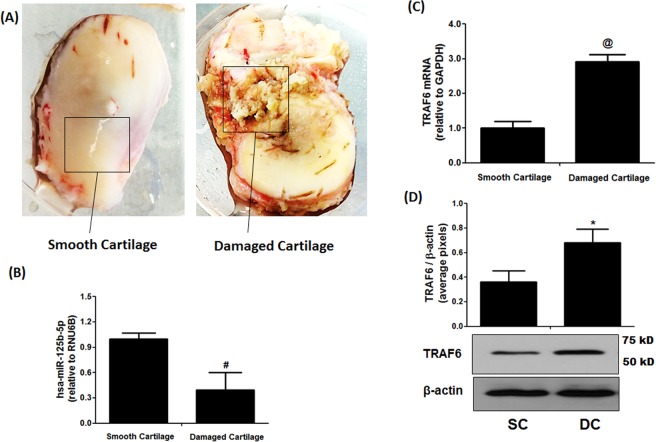
(**A**) MicroRNA database top predicted targets for microRNA hsa-miR-125b-5p. The number in the center of each circle represents target score of prediction. Full names of all genes are available at https://www.ncbi.nlm.nih.gov/gene. (**B**,**C**) Bioinformatics analysis of hsa-miR-125b-5p binding to 3′UTR of TRAF6 mRNA. (**B**) Seed-Matched sequences of hsa-miR-125b-5p in the 3′UTR of TRAF6 mRNA. (**C**) Target Scan prediction. **(D)** Cross-species conservation by Target Scan algorithm.

We used taqman assays to determine the expressions of hsa-miR-125b-5p and TRAF6 in RNA samples directly isolated from damaged and smooth OA cartilage and the results are presented in Fig. 2. The taqman assays showed inverse co-relation between hsa-miR-125b-5p and TRAF6 expression in both damaged and smooth cartilage tissues (Fig. 2B,C). These results were further verified by protein expression of TRAF6 determined by western immunoblotting (Fig. 2D). As shown in Fig. 2D, TRAF6 at protein level in the damaged cartilage was significantly higher than the level present in the smooth cartilage. This negative co-relation of hsa-miR-125b-5p and TRAF6 in damaged or smooth cartilage indicates a potential role of hsa-miR-125b-5p in the pathogenesis of OA.

### Expression of hsa-miR-125b-5p and TRAF6 in human OA chondrocytes

**Figure 3:**
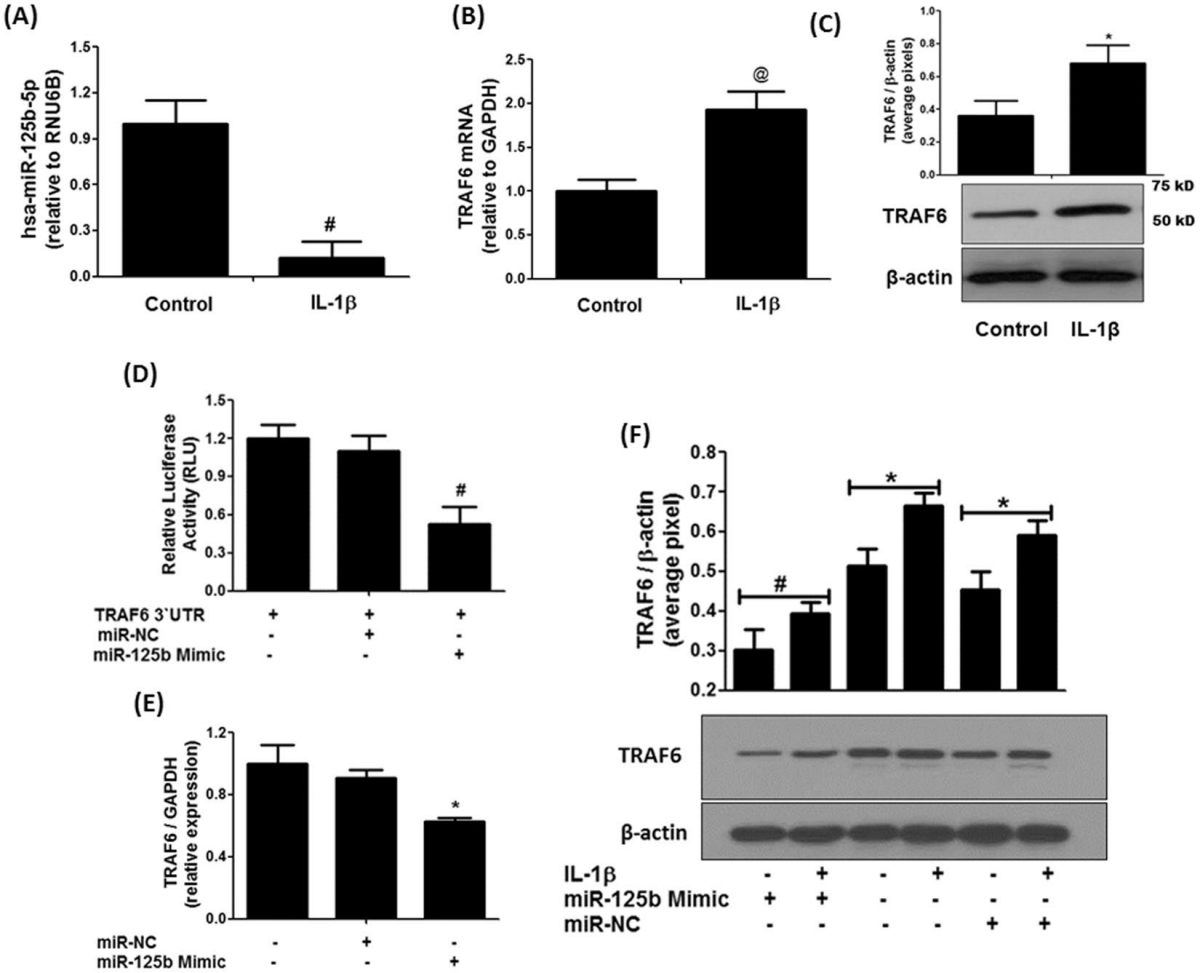
Inverse co-relation between hsa-miR-125b-5p and TRAF6 and validation of hsa-miR-125b-5p binding to 3′UTR of TRAF6 mRNA in human OA chondrocytes. (**A**) Expression of hsa-miR-125b-5p in IL-1β-stimulated human OA chondrocytes. ^#^p < 0.01 versus control (**B**) Gene expression of TRAF6 in IL-1β-stimulated human OA chondrocytes. ^@^p < 0.05 versus control (**C**) Protein expression of TRAF-6 in IL-1β-stimulated human OA chondrocytes (full length blot is provided in Supplementary Information File). ^*^p < 0.01 versus control. (**D**) Luciferase activity in transfected OA chondrocytes with the reporter vector and miR-125b-5p mimic. ^#^p < 0.05 versus TRAF6 3′UTR alone, ^#^p < 0.05 versus TRAF6 3′UTR + miR-NC (**E**) Gene expression of TRAF6 in miR-125b mimic transfected OA chondrocytes stimulated with IL-1β. ^*^p < 0.05 versus miR-NC alone, ^#^p < 0.05 versus untreated chondrocytes (**F**) Expression of TRAF6 protein in miR-125b-5p transfected OA chondrocytes stimulated with IL-1β (full length blot is provided in Supplementary Information File). ^#^p < 0.05 versus *. Band images were digitally captured by the Un-Scan-It software and the band intensities of TRAF6 proteins were divided by band intensities of β-actin and are expressed in average pixels. Unstimulated chondrocytes were used as controls and expression of RNU6B/GAPDH was used as an endogenous control.

Treatment of cartilage cells with IL-1β for 40 minutes, significantly inhibited hsa-miR-125b-5p expression (Fig. 3A) and significantly increased the expression of TRAF6 mRNA (Fig. 3B). To validate TRAF6 mRNA results, we also determined protein expression of TRAF6 in the chondrocytes lysates stimulated with same amount of IL-1β and the results were same as we found at mRNA level (Fig. 3C). The altered protein expression of TRAF6 might be related with the inverse correlation of hsa-miR-125b-5p and TRAF6 gene ratio.

### MicroRNA-125b-5p binds to 3′UTR of TRAF6 mRNA

To validate the recognition of 3′UTR of TRAF6 by hsa-miR-125b-5p, we performed transfection experiment with the reporter clone contained whole 3′UTR of TRAF6 mRNA (NM_004620.3) and the results are presented in Fig. 3D. The co-transfection of cells with 3′UTR TRAF6 clone and miR-125b mimic significantly reduced the luciferase activity as compared to those sets of cells transfected with 3′UTR TRAF6 clone and miR-NC (Fig. 3D). These results validated the recognition of 3′UTR of TRAF6 by hsa-miR-125b-5p. To further validate this recognition between the two, the cells were transfected with miR-125b mimic (or miR-NC) and then treated with IL-1β. Our results showed that transfection with miR125b mimic showed significant inhibition of IL-1β-induced gene and protein expression of TRAF6 (Fig. 3E,F). These results confirmed that hsa-miR-125b-5p directly regulates the expression and production of TRAF6 via its 3′UTR in human OA chondrocytes.

### Effect of hsa-miR-125b-5p on the IL-1β-induced activation of p38, JNK, ERK -MAPKs

**Figure 4:**
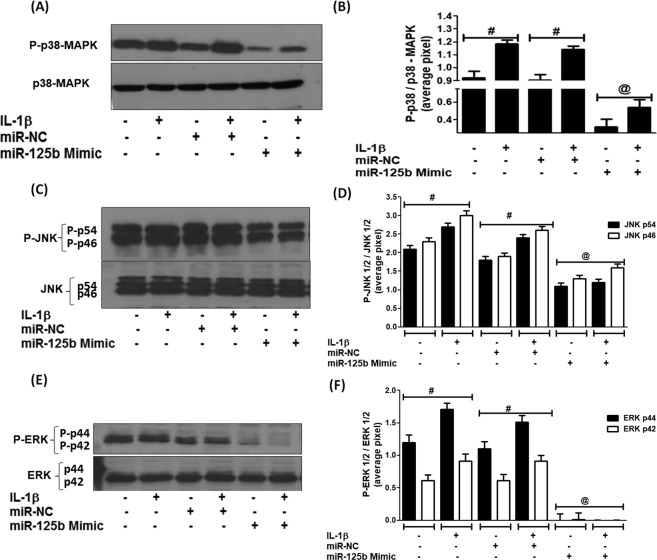
MicroRNA hsa-miR-125b-5p regulates MAPKs in human OA chondrocytes. (**A**,**B**) Inhibition of p38-MAPK signaling in miR-125b mimic transfected human chondrocytes (full length blot is provided in Supplementary Information File). ^@^p < 0.01 versus #. (**C**,**D**) Inhibition of JNK signaling in miR-125b mimic transfected human chondrocytes (full length blot is provided in Supplementary Information File). ^@^p < 0.05 versus #. (**E**,**F**) Inhibition of ERK signaling in miR-125b mimic transfected human chondrocytes (full length blot is provided in Supplementary Information File). ^@^p < 0.001 versus #. Unstimulated chondrocytes were used as controls and expression of RNU6B/GAPDH was used as an endogenous control. Band images were digitally captured by the Un-Scan-It software and the band intensities of phosphorylated proteins were divided by band intensities of total proteins and are expressed in average pixels.

To investigate whether p38-MAPK, JNK-MAPK or ERK-MAPK are involved in IL-1β-induced down-regulation of hsa-miR-125b-5p in human OA chondrocytes, we transfected cells with miR-125b mimic and then stimulated with IL-1β for 30 minutes and the cell lysates were analyzed by western immunoblotting using specific primary antibodies for MAPKs (Cell Signaling Technology). Our data showed that the transfection of cells with miR-125b mimic caused noticeable reduction in p38-MAPK phosphorylation in both sets of cells either stimulated and non-stimulated (Fig. 4A,B). Our data also showed that the transfection of cells with miR-125b mimic also caused significant inhibition in the phosphorylation of both JNK-MAPKs subtypes p54 and p46 and also caused marked reduction in the phosphorylation of ERK-MAPK subtype’s p44 and p42 in both stimulated and non-stimulated cells (Fig. 4E,F). Whereas transfection with pre-miR-NC showed insignificant effect on the phosphorylation of all studied MAPKs. These findings clearly indicate that hsa-miR-125b-5p inhibits the activation of p38, JNK, or ERK –MAPKs in the studied cartilage cells.

### Expression of hsa-miR-125b-5p and NF-κB activation

**Figure 5:**
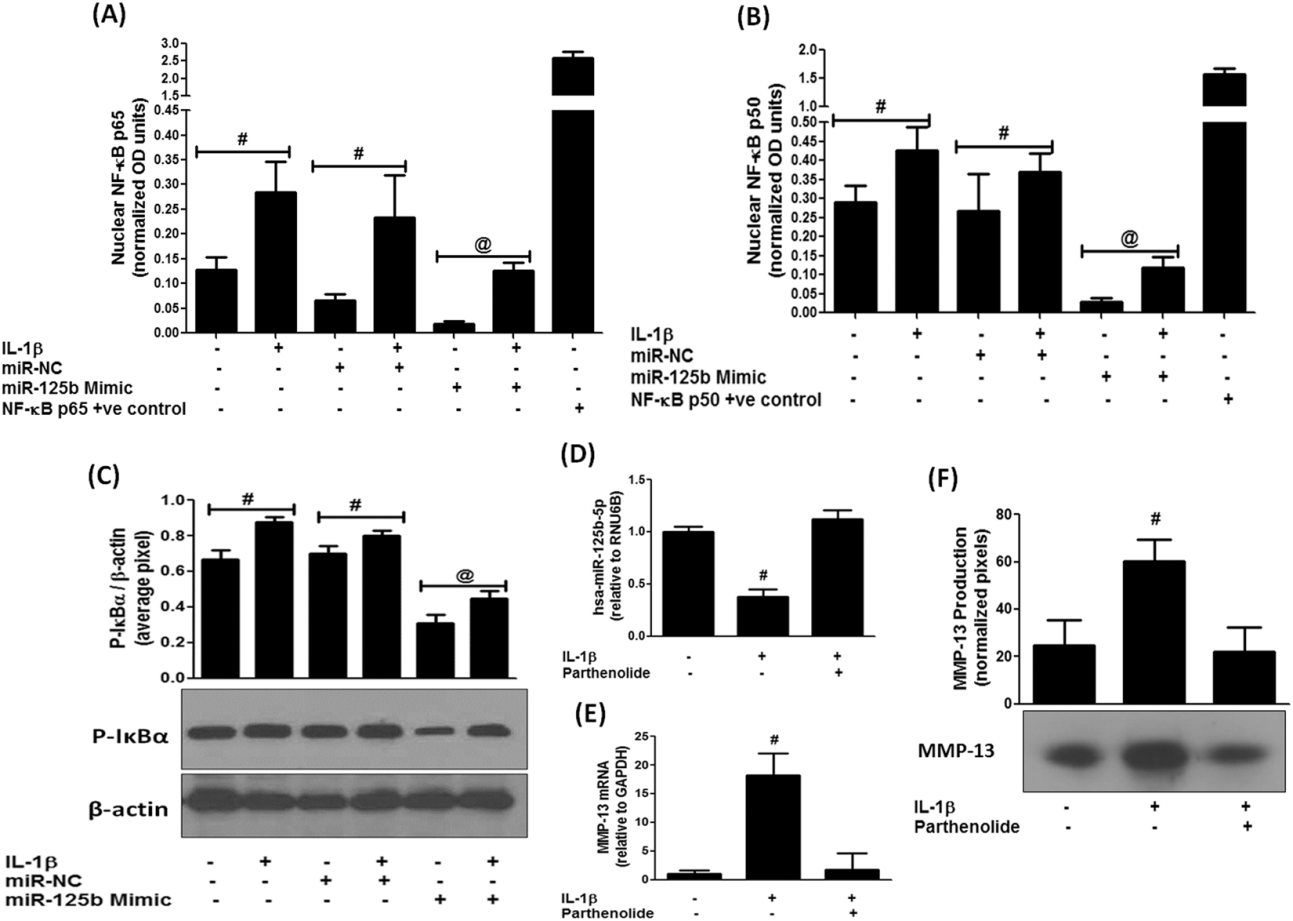
MicroRNA hsa-miR-125b-5p regulates NF-κB signaling events in human OA chondrocytes. (**A**) Inhibition of NF-κB p65 activity in miR-125b mimic transfected human chondrocytes. ^@^p < 0.01 versus #. (**B**) Inhibition of NF-κB p50 activity in miR-125b mimic transfected human chondrocytes. ^@^p < 0.01 versus #. (**C**) Inhibition of IκBα activation in miR-125b mimic transfected human chondrocytes (full length blot is provided in Supplementary Information File). ^@^p < 0.05 versus #. (**D**–**F**) Parthenolide (IKK inhibitor) negatively co-regulated hsa-miR-125b-5p expression (**D**) and MMP-13 mRNA expression (**E**) and production (**F**) in IL-1β-stimulated human chondrocytes ^#^p < 0.05 versus parthenolide treated chondrocytes. Unstimulated chondrocytes were used as controls and expression of RNU6B/GAPDH was used as an endogenous control. Band images were digitally captured and the band intensities (pixels/band) were obtained using the Un-Scan-It software and are expressed in average pixels.

To investigate whether activation of nuclear transcription factor NF-κB is required for the down-regulation of hsa-miR-125b-5p expression in human OA chondrocytes, we transfected cells with miR-125b mimic (or pre-miR-NC) and then stimulated with IL-1β for 30 minutes and the levels of nuclear NF-κBp50 and NF-κBp65 were determined by the NF-κB p50/p65 Transcription Factor Binding Assay ELISA kit (Abcam). Our data showed that the transfection of cells with miR-125b mimic markedly reduced the nuclear levels of NF-κBp65 in IL-1β-stimulated cartilage cells (Fig. 5A). Whereas transfection with pre-miR-NC showed insignificant effect on the nuclear levels of NF-κBp65 (p > 0.05). Moreover, same sets of transfected cells also significantly reduced the nuclear levels of NF-κBp50 upon IL-1β stimulation or non-stimulation (p < 0.01). Whereas, transfection with pre-miR-NC showed insignificant effect on the nuclear levels of NF-κBp50 (Fig. 5B). Furthermore, our data also showed the destabilization of IκBα in the cytoplasm of these transfected cells. As transfection with miR-125b mimic showed significant reduction in the phosphorylation of IκBα in both stimulated and non-stimulated cells (Fig. 5C). However, transfection with pre-miR-NC had no effect on the phosphorylation of IκBα levels (p > 0.05). To investigate more on the involvement of NF-κB signaling in the regulation of microRNA hsa-miR-125b-5p, we treated human OA chondrocytes with IκB kinase inhibitor parthenolide. As shown in Fig. 5D–F, treatment with parthenolide significantly up-regulated hsa-miR-125b-5p expression (Fig. 5D) and significantly inhibited the MMP-13 mRNA expression (Fig. 5E) or MMP-13 protein secretion in the culture medium (Fig. 5F). These results clearly indicate that the activation of NF-κB is negatively co-related with hsa-miR-125b-5p expression in human OA chondrocytes.

### Anti-inflammatory activity of hsa-miR-125b-5p on MMP-13

**Figure 6:**
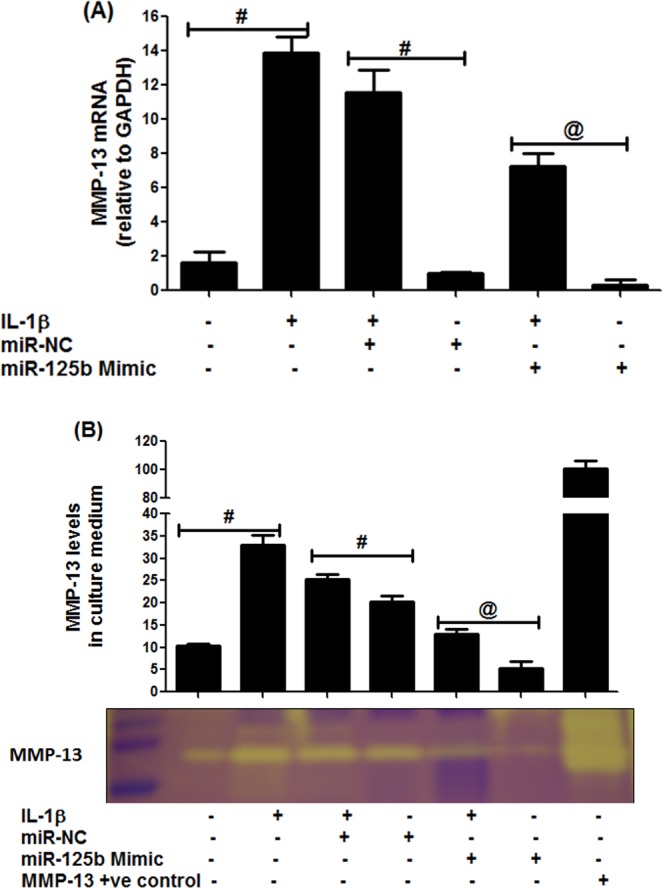
MicroRNA hsa-miR-125b-5p regulates MMP-13 expression in human OA chondrocytes. (**A**) MMP-13 gene expression in IL-1β stimulated chondrocytes transfected with miR-125b mimic. ^@^p < 0.01 versus #. (**B**) MMP-13 activity in the culture supernatant in OA chondrocytes transfected with miR-125b mimic. The production of MMP-13 was determined in cell culture supernatant by gelatin zymography (full length zymographic expression is provided in Supplementary Information File). Equal volumes of culture supernatant were loaded on polyacrylamide gel. The MMP-13 positive control (EMD Chemicals) was also used. Band images were digitally captured and the band intensities (pixels/band) were obtained using the Un-Scan-It software and are expressed in average pixels. Unstimulated chondrocytes were used as controls and expression of RNU6B/GAPDH was used as an endogenous control.

To analyze anti-inflammatory activity of hsa-miR-125b-5p on the overproduction of MMP-13 in human OA chondrocytes, the cells were transfected with miR-125b mimic (or pre-miR-NC) and were then stimulated with IL-1β for 24 hours. Results in Fig. 6A showed that the transfection with miR-125b mimic significantly inhibited gene expression of MMP-13 in stimulated and non-stimulated cells (Fig. 6A). Results also showed that the transfection of same cells with pre-miR-NC or non-transfected cells had no effect on the expression of MMP-13 (Fig. 6A). Not only have these, we also determine whether inhibition of MMP-13 gene expression in miR-125b mimic transfected chondrocytes also affected on its production, the culture medium was analyzed for MMP-13 by zymography. Results in Fig. 6B showed that transfection of OA cells with miR-125b mimic significantly suppressed MMP-13 secretion in both stimulated and non-stimulated cells (p < 0.05). Whereas, transfection with pre-miR-NC had no effect on MMP-13 secretion on the same sets of cells (p > 0.05). These findings confirmed that hsa-miR-125b-5p inhibits IL-1β-induced MMP-13 in human OA chondrocytes.

### Impact of hsa-miR-125b-5p on IL-1β-induced secretion of cytokines, chemokines and growth factors

**Figure 7:**
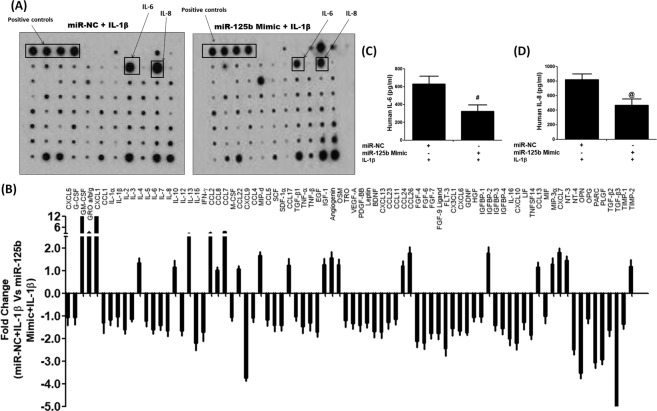
Impact of hsa-miR-125b-5p on the production of cytokines in human OA chondrocytes. (**A**) Human cytokine antibody array was used to quantify the production of 80 cytokines in the pooled cultured medium (n = 5) from the transfected OA chondrocytes with miR-125b mimic or miR-NC and stimulated with IL-1β for 24 hours. (**B**) Densitometry analysis of spots images digitally captured using the Un-Scan-It software and expressed as average pixels of five shots. (**C**) Fold change in the spot intensities of the IL-1β-stimulated human OA transfected chondrocytes with miR-NC versus transfected chondrocytes with miR-125b mimic. (**D**) Human OA chondrocytes (n = 5) were transfected with miR-125b mimic and then stimulated with IL-1β for 24 hours to quantify IL-6 production using IL-6 specific Sandwich ELISA (Genway). ^#^p < 0.05 versus miR-NC + IL-1β. (**F**) Human OA chondrocytes (n = 5) were transfected with miR-125b mimic and then stimulated with IL-1β for 24 hours to quantify IL-8 production using IL-8 specific Sandwich ELISA (Genway). ^@^p < 0.05 versus miR-NC + IL-1β.

**Figure 8:**
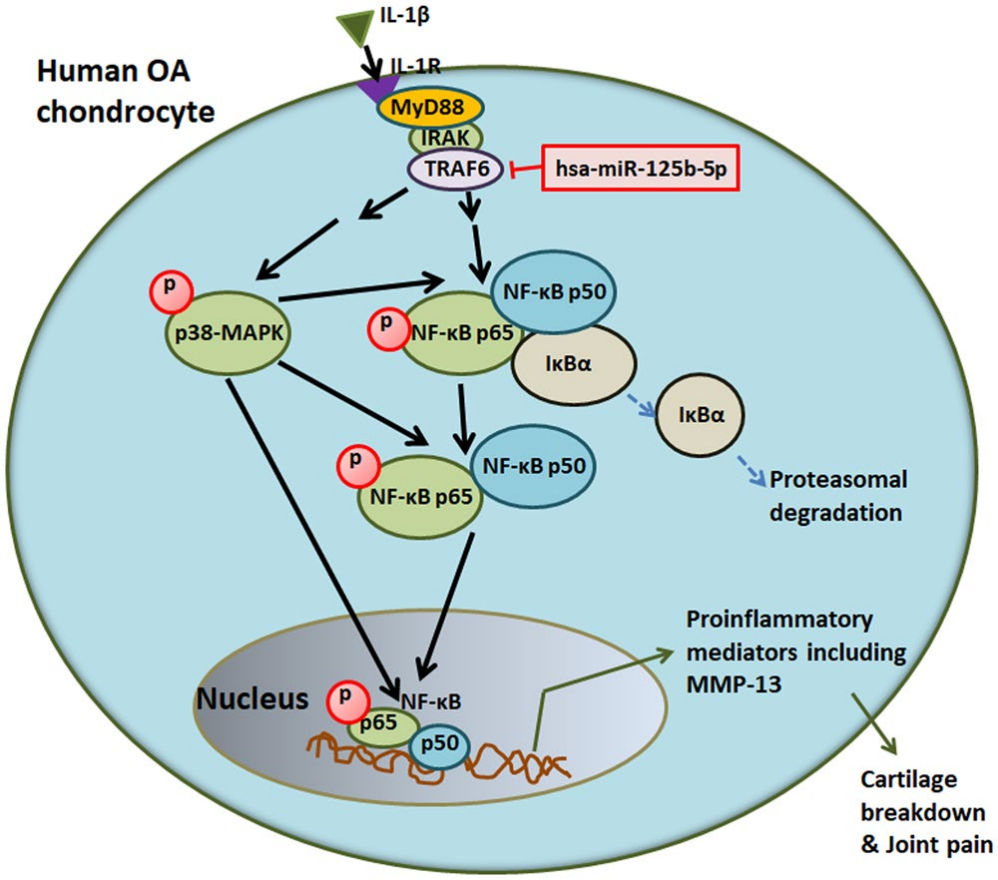
Anti-inflammatory action of hsa-miR-125b-5p against IL-1β induced inflammatory signaling in human OA chondrocytes. IL-1β through IL-1R activates TRAF6 mediated signaling to the nuclear transcription factor NF-κB via activation of MARKs induced production of inflammatory mediators including MMP-13 in human OA chondrocytes. MicroRNA-125b-5p regulates MMP-13 expression via TRAF6 3′UTR. Abbreviations: OA: osteoarthritis; IL-1β: interleukin-1 beta; IL-1R, IL-1 receptor; TRAF6: TNF receptor associated factor 6; 3′UTR: three prime untranslated region; MAPK: mitogen activated protein kinase; NF-κB: nuclear transcription factor; IκBα: inhibitor of kappa B protein; MMPs: matrix metalloproteinases.

We used cytokine array to determine the effect of hsa-miR-125b-5p on IL-1β-induced secretion of proinflammatory cytokines, chemokines and growth factors and the results are shown in Fig. 7A. The results obtained from the densitometry analysis of the protein array membranes of cultured medium of cells transfected with pre-miR-NC or miR-125b mimic in the presence of IL-1β showed marked suppression in the secretion of several proinflammatory cytokines, chemokines and growth factors which are known to contribute to arthritis including IL-6 (1.59 folds), IL-7 (1.40 folds), IL-8 (1.65 folds), IL-15 (2.2 folds), IL-12 (1.65 folds), INF-γ (1.71 folds), TNF-α (1.45 folds), TGF-β1 (1.02 folds), TGF-β2 (1.63 folds), TGF-β3 (5.6 folds), IGFBP-1 (1.02 folds), IGFBP-3 (1.41 folds), IGFBP-4 (1.54 folds), FGF-4 (2.19 folds), FGF-6 (2.20 folds), FGF-7 (1.75 folds), FGF-9 ligand (1.77 folds), PDGF-BB (1.39 folds) and VEGF (1.33 folds) (Fig. 6B). In contrast, few cytokines, chemokines or growth factors were found to be overproduced in the same culture medium of transfected cells such as IL-10, IL-13, CXCL1, IGF-1 and the rest were shown in Fig. 6B. To validate these protein array results, we used the same culture medium to quantitate IL-6 and IL-8 secretion by their specific sandwich ELISAs (Genway Biotech Inc., ELISA Kits, CA, USA). Protein arrays results were consistent as the transfection with miR-125b mimic caused significant suppression of IL-1β-induced IL-6 and IL-8 production (Fig. 7C,D). Diagrammatic representation of anti-inflammatory action of hsa-miR-125b-5p against IL-1β induced inflammatory signaling in human OA chondrocytes is summarized in Fig. 8.

## Discussion

This is the first study demonstrated that microRNA hsa-miR-125b-5p regulates inflammatory genes including MMP-13 via targeting TRAF6 mediated MAPKs and NF-κB pathways in human OA chondrocytes. It is now well documented that overproduction of IL-1β or MMP-13 in arthritic joints are most powerful cartilage damaging factors for the onset and progression of OA^25,26^. IL-1β-mediated MMP-13 overproduction in the joints are well known to induce cartilage breakdown and for the induction of arthritic pain^27^ and are now considered as a hallmark of OA development^25–27^. Recent studies pointed out the role of miRNAs in OA pathology, which are assumed to play a role in cartilage breakdown through pairing with genes relevant to OA pathology including proinflammatory cytokines, chemokines, or some growth factors^9–14,23^ and now we believe that targeting of specific miRNAs is a powerful approach for OA management. In this study, we show the involvement of microRNA-125b-5p in regulation of several inflammatory genes including MMP-13 in human OA chondrocytes. MicroRNA-125 has highly conserved nucleic acid sequence reported in several mammals^28,29^. In humans, it is basically found in three homologs structures hsa-miR-125a, hsa-miR-125b-1 and hsa-miR-125b-2 and the mature hsa-miR-125b-5p was derived from the pre-mature hsa-miR-125a, hsa-miR-125b-1^28,29^. Recently, the role of miR-125b was discovered in various cell types of different human disorders^16–21^. More specifically, miR-125b was reported to inhibit keratinocyte proliferation and promote their apoptosis via targeting PI3K/Akt/mTOR pathway and regulates expression of MMP-2^30^. Moreover, its reduced expression was found in squamous cells of oral cancer patients, suggesting its direct role in oral cancer^31^. Furthermore, its abnormal expression was reported in different organs of different cancer patients^17^, suggesting its role in cancer. Now, the role miR-125b is well established in regulation of cancers associated genes, therefore it is presumed that cancer associated mechanisms may have its involvement. Importantly, the role of miR-125b was also reported in regulation of key inflammatory genes in different cell types, suggesting its role in inflammation^20,21,32^. Furthermore, its role was also reported in acute chronic pulmonary disorders^33^. Not only have these, miR-125b also promotes activation of macrophages, suggesting its role in immune system^34^. In OA, the role of miR-125b was defined by few studies, in one study miR-125b was reported to regulate aggrecanase-1 expression in human chondrocytes^20^ and in another study, its role was defined in regulation of inflammatory activities in stimulated chondrogenic cells^21^. Moreover, in one of our previous studies, using microarray analysis, we analyzed the expression of 1347 miRNAs in human OA chondrocytes stimulated with IL-1β. Out of them, 35 miRNAs were differentially expressed upon IL-1β stimulation and among differentially expressed miRNAs, hsa-miR125b-5p was found to be down-regulated by more than 100 folds^12^. In view of this interesting finding and findings by the others, the present study was designed to investigate the role of hsa-miR-125b-5p in modulation of inflammatory genes relevant to OA pathogenesis. Our results show that the hsa-miR125b-5p negatively co-regulates the expression of TRAF6 through direct binding with its 3′UTR in primary OA chondrocytes. Using microRNA database bioinformatics approach at this locus, 926 genes was found that showed sequences similar to the seed sequence of hsa-miR125b-5p. Among them, the predicted scores of binding between the hsa-miR125b-5p and TRAF6 mRNA sequence was 85, indicating strong chances of binding between them. These computer-based predictions were further re-confirmed by another bioinformatics approach. Applying targetscan algorithm, we identified that the sequence of hsa-miR-125b-5p is complementary to 3′UTR of human TRAF6 mRNA at 1276–1283 nucleotide sequence. Not only in human TRAF6 mRNA, similar complementary sequence was also found in the TRAF6 3′UTR of chimpanzee, rhesus, mouse, rat, cat, dog and elephant. To prove these computer based predictions, taqman assays were used to determine the expression of hsa-miR-125b-5p and TRAF6 in OA cartilage obtained from discarded knee/hips tissues from OA patients. Taqman assays pointed out that the expression of hsa-miR-125b-5p was significantly more in smooth OA cartilage as compared to its expression in damaged OA cartilage. Whereas, the expression of TRAF6 in damaged cartilage was significantly high as compared to smooth cartilage. This inverse correlation between hsa-miR-125b-5p and TRAF6 in damaged and smooth OA cartilage may indicate that hsa-miR-125b-5p may regulates TRAF6 expression in OA cartilage. As we know chondrocytes are the only cell types of the cartilage^35^, therefore taqman assays specific for hsa-miR-125b-5p and TRAF6 were applied to determine their expression in chondrocytes obtained from OA cartilage. Again the same correlation between hsa-miR-125b-5p and TRAF6 was found in both sets of stimulated or unstimulated cells. However, treatment of cells with IL-1β remarkably increased this negative co-relation, suggesting involvement of one or more mechanisms in human OA chondrocytes with or without IL-1β. Earlier investigations proved that the IL-1β induces cartilage breakdown by altering the inflammatory mechanisms^4,36^. In support of these, our data clearly indicate that IL-1β stimulates the proinflammatory mechanisms by inhibiting hsa-miR-125b-5p expression and stimulating TRAF6 mediated cell signaling which promote joints breakdown in OA. To validate the direct binding between 3′UTR of TRAF6 mRNA and hsa-miR-125b-5p, we performed luciferase reporter assays. A noticeable inhibition in the luciferase activity was found in cells with overexpressed hsa-miR-125b-5p. Furthermore, treatment with miR-125b mimic caused significant suppression of IL-1β induced TRAF6 expression. These results validated that hsa-miR-125b-5p regulates the expression of TRAF6 through its 3′UTR in human OA chondrocytes.

The MAPKs are key inflammatory pathways associated with OA pathogenesis and IL-1β induced activation of MAPKs via TRAF6 was well studied in human OA chondrocytes^37–39^. Furthermore, involvement of MAPKs in regulation of miRNAs expression was also testified^40^. In the present study, we show that the hsa-miR-125b-5p regulates not only p38-MAPK, but also JNK and ERK-MAPKs in chondrocytes stimulated with IL-1β. Treatment of chondrocytes with miR-125b mimic caused marked inhibition in the phosphorylation of all three studied MAPKs, indicating that altered expression of hsa-miR-125b-5p is regulated by MAPKs activation. The master transcription factor NF-κB is the most studied transcription factors associated with inflammation^38,41^. It is now well known that IL-1β alters inflammation through NF-κB pathway^42,43^ and activation of NF-κB is also reported to involve in miRNAs expression^44^. Importantly it is also reported that IL-1β induced phosphorylation of TRAF6 and MAPKs also activates the NF-κB event^39^. In this study, we determined inhibition of NF-κB destabilization in the cytoplasm of the stimulated cells by the altered expression of hsa-miR-125b-5p. As transfection of cells with miR-125b mimic significantly inhibited the IL-1β-induced the nuclear translocation of NF-κBp65 and NF-κBp50 and also significantly phosphorylation of IκBα in these transfected cells. These results confirmed the involvement of NF-κB in the hsa-miR-125b-5p regulation in these studied cartilage cells.

Evidences have shown that IL-1β and MMP-13 are present in the higher levels in the sites of damaged OA cartilage tissue, indicating their direct involvement in the OA pathogenesis^45,46^, therefore the involvement of hsa-miR-125b-5p in the IL-1β-induced overproduction MMP-13 was investigated. Our findings showed that the treatment of cells with miR-125b mimic caused remarkable reduction of MMP-13 expression in the studied cartilage cells, suggesting an anti-inflammatory activity of hsa-miR-125b-5p on overproduction of MMP-13 in the OA. To further understand the impact of inhibition of IL-1β-induced TRAF-6/MAPKs/NF-κB mediated signaling by hsa-miR-125b-5p, we determined the effect of hsa-miR-125b-5p on the IL-1β induced overproduction of 80 different inflammatory mediators including inflammatory cytokines, chemokines, growth factors by cytokine arrays. Data show that treatment of chondrocytes with miR-125b mimic caused marked inhibition in IL-1β-induced secretion of number of proinflammatory cytokines, chemokines and growth factors which are relevant to OA pathogenesis including IL-6, IL-15, TNF-α, INF-γ, IL-8, IGF-β1, TGF-β2 and IGFBP-1^5,6,24,47,48^. These interesting results further strengthen our central hypothesis that hsa-miR-125b-5p plays a key role in negative co-regulation of genes involved in the destruction of joints in OA. Taking all together, these findings demonstrate that the hsa-miR-125b-5p regulates inflammatory genes including MMP-13 via targeting TRAF6 mediated MAPKs and NF-κB signaling events in human OA chondrocytes.

## Conclusions

This is the first report shows that the hsa-miR-125b-5p regulates several inflammatory genes including MMP-13 via direct binding with 3′UTR of TRAF6 mRNA in human OA chondrocytes. Expression of hsa-miR-125b-5p is negatively co-related with TRAF6/MAPKs/NF-κB pathways. In short, the study concludes that targeting 3′UTR of TRAF6 or hsa-miR-125b-5p may be useful for therapeutic application for the prevention of cartilage breakdown in OA.

## Methods

### Articular chondrocytes culture preparation

The present study was carried out in accordance with Code of Ethics of World Medical Association (Declaration of Helsinki as revised in Tokyo 2004) for humans and was approved by Ministry of Health, KSA (IRB Registration # with KACST, KSA: H-01-R-012; IRB Registration # with OHRP/NIH, USA: IRB00008644; Approval Number Federal Wide Assurance NIH, USA: FWA00018774). Informed consent was obtained from all participants. With the approval of Ministry of Health and from the involved participants, discarded knee or hip tissues were obtained from 17 OA patients (mean ± SD age: 74.7 ± 10.4 years), who went for joint replacement surgery at King Fahd Medical City, KSA. The macroscopic cartilage degeneration was determined by staining of femoral head samples with India ink and the cartilage with smooth articular surface (“unaffected cartilage”) was only resected for the preparation of chondrocytes after digestion with pronase and collagenase (Roche Diagnostics, Mannheim, Germany) as described previously^49^. The used smooth cartilage was anatomically located in the peripheral (“marginal”) part of the discarded knee or hips tissues. Texture of damaged cartilage from OA knee or hip tissues was also analyzed and was shown cartilage erosion on the medial humeral condyle, ulceration of cartilage was found in the medial femoral condyle. Moreover, osteophytes formation was also seen in the OA tissue samples. The area selected on the cartilage tissues for the preparation of chondrocytes are shown in Supplementary Fig. 1. Isolated cartilage cells were plated at a density of 1.2 × 10^6^/ml in complete DMEM medium as previously described^50^.

### Stimulation of primary human OA chondrocytes and preparation of microRNAs

Human OA chondrocytes (1.2 × 10^6^/well) were serum-starved for 12 hours/overnight and were treated with IL-1β (5 ng/ml; catalog # IL038, EMD Millipore corporation, Temecula, CA, USA)^13,51^. We used mirVana miRNA isolation kit (catalog # AM1560, Ambion, CA, USA) for the isolation of miRNA fraction. For some experiments, total RNA was prepared directly from cartilage as described previously^13^. Briefly, cartilage was sliced off from discarded knee or hip tissues of OA patients and RNA then was purified according to the manufacturers’ instructions (Ambion, CA, USA).

### Quantitative PCR analysis

Total RNA (04–0.8 µg) was first reverse-transcribed by SuperScript First Strand cDNA synthesis kit (catalog # 75780, Affymetrix Inc., OH, USA) and then the expression of TRAF6 or MMP-13 mRNA or hsa-miR-125b-5p was measured using the TaqMan Gene Expression Assays (Applied Biosystems, Foster City, CA) as described previously^13^. Quantitative PCR amplification and data capture were carried out through the Step One Real Time PCR System (Applied Biosystems). Relative expression levels were analyzed using ΔΔCT method^52^.

### Western immunoblotting

Protein expression in human OA chondrocytes was analyzed by western immunoblotting as described previously^53^. Band images were digitally captured using the UN-SCAN-IT (Silk Scientific Corporation, Utah, USA) and each band was scanned 5 times with background correction and values were expressed as average pixel band ratios as described previously^13^.

### Gelatin zymography for MMP-13 determination

Gelatin zymography was performed as described previously^27^. Briefly, after gel electrophoresis of the culture medium, the gel containing 0.2% gelatin was digested using the gelatinase buffer at 37 °C for 18 hours and was stained with Coomassie brilliant blue R350 and the locations of gelatinolytic activity were revealed as clear bands on a background of uniform light blue staining. MMP-13 on the gel was analyzed by the electrophoretic migration of the recombinant human MMP13 protein (catalog #: ab134452, Abcam, Cambridge, MA, USA) and also by the known molecular weight standards (Catalog # 161–0399, Precisian plus protein Western Standards, Bio-Rad, USA). After digestion, clear band images were analyzed using Un-Scan-It software (Silk Scientific Corporation).

### Luciferase reporter assay

Human OA chondrocytes were co-transfected with luciferase reporter plasmids together with miR-125b mimic (Qiagen, Valencia, USA or Ambion, Austin, Texas, USA) using HiPerfect Transfection Reagent (Qiagen) as described previously^13^. Dual-Luciferase Reporter Assay System (Promega, Madison, WI, USA) was used to analyze the luciferase activity after 24 hours post-transfection. Relative luciferase activity was measured by the ratio of reporter (firefly) to control (renilla) activity as instructed by the manufacturers (Promega).

### Transfection with pre-miRNAs

Human OA chondrocytes were transfected with pre-miRNAs (100 nM; Ambion/Qiagen) at a 100 nM concentration, using the calcium phosphate precipitation method^13,54^. Following transfection, cells were treated with IL-1β (5 ng/ml) for different time intervals to analyze the desire mRNA expression.

### Preparation of nuclear extracts and determination of NF-κB activity

To study the effect of hsa-miR-125b-5p on IL-1β-induced NF-κBp50 or NF-κBpp65, OA chondrocytes were transfected with miR-125b mimic or pre-miR-NC (100 nM) and then stimulated or not with IL-1β (5 ng/ml) for 30 minutes. Upon termination of the reaction, chondrocytes were washed with ice-cold PBS and then scraped and centrifuged at 1500 × g for 5 minute at 4 °C. Nuclear factions were prepared as described previously^55^ and equal amount of protein from nuclear extracts were used for the activation or destabilization of NF-κBp50 and NF-κBp65 using a highly sensitive and specific Transcription Factor ELISA Kit according to the manufacturer instructions (catalog # ab133128, Abcam, MA, USA).

### Cytokine antibody array

A commercial human cytokine antibody-based array was used to determine the production of cytokines, chemokines and growth factors (RayBio Human Cytokine Array C5, RayBiotech, GA, USA). Human OA chondrocytes were transfected with miR-125b mimic or miR-NC for 48 h, followed by IL-1β treatment for 24 hours. The culture medium from five OA patients was pooled and used in the array experiment as per recommendations mentioned by the manufacturer. Spots obtained were analyzed using Un-Scan-It software (Silk Scientific Corporation) and mean of five individual readings was considered.

### Statistical analysis

We used Graph Pad Prism-5 (San Diego, CA, USA) for statistical analysis and the data were compared by One-way ANOVA analysis followed by Tukey’s post-hoc analysis or Two-way ANOVA followed by Bonferroni post-hoc tests and p < 0.05 was considered significant.

## Supplementary information

Supplementary Data

## Data Availability

The datasets generated and/or analyzed during this study are available with corresponding author and will be sent on reasonable request.
