# Author Correction: MicroRNA-125b-5p regulates IL-1β induced inflammatory genes via targeting TRAF6-mediated MAPKs and NF-κB signaling in human osteoarthritic chondrocytes

**DOI:** 10.1038/s41598-019-50844-3

**Published:** 2019-10-08

**Authors:** Zafar Rasheed, Naila Rasheed, Waleed Al Abdulmonem, Muhammad Ismail Khan

**Affiliations:** 1 Department of Medical Biochemistry, College of Medicine, Buraidah, Qassim University, Buraidah, Saudi Arabia; 2 Department of Pathology, College of Medicine, Buraidah, Qassim University, Buraidah, Saudi Arabia; 3 Faculty of Medicine, School of Public Health, University of Queensland, Brisbane, Australia

Correction to: *Scientific Reports* 10.1038/s41598-019-42601-3, published online 03 May 2019

This Article contains an error in the order of the Figure legends. The legends for Figures 1 and 2 were incorrectly given as those for 2 and 1 respectively.

The legend for Figure 1 should read:

“**(A)** MicroRNA database top predicted targets for microRNA hsa-miR-125b-5p. The number in the center of each circle represents target score of prediction. Full names of all genes are available at https://www.ncbi.nlm.nih.gov/gene. (**B**,**C**) Bioinformatics analysis of hsa-miR-125b-5p binding to 3′UTR of TRAF6 mRNA. **(B)** Seed-Matched sequences of hsa-miR-125b-5p in the 3′UTR of TRAF6 mRNA. **(C)** Target Scan prediction. **(D)** Cross-species conservation by Target Scan algorithm.”

The legend for Figure 2 should read:

“Expression of hsa-miR-125b-5p and TRAF6 in smooth and damaged OA cartilage tissues. **(A)** Smooth and damaged cartilage tissues from OA patients **(B)** Expression of hsa-mir-125b-5p in smooth and damaged cartilage tissues. ^#^p < 0.01 versus smooth cartilage tissue. **(C)** Gene Expression of TRAF6 in smooth and damaged cartilage tissues. ^@^p < 0.01 versus smooth cartilage tissue. **(D)** Protein expression of TRAF6 in smooth and damaged cartilage tissues (full length blot is provided in Supplementary Information File). *p < 0.01 versus SC. SC, smooth cartilage; DC, damaged cartilage. Band images were digitally captured by the Un-Scan-It software and the band intensities of TRAF6 proteins were divided by band intensities of β-actin and are expressed in average pixels.”

