# Supplementary material for: MicroRNA-125b-5p regulates IL-1β induced inflammatory genes via targeting TRAF6-mediated MAPKs and NF-κB signaling in human osteoarthritic chondrocytes
Source: Sci Rep. 2019 May 3;9:6882. doi: 10.1038/s41598-019-42601-3 (PMC6499837; doi:10.1038/s41598-019-42601-3)

MicroRNA-125-5p regulates IL-1β induced inflammatory genes via targeting TRAF6-mediated MAPKs and NF-κB signaling in human osteoarthritic chondrocytes

Zafar Rasheed[1,*], Naila Rasheed[1], Waleed Al Abdulmonem[2], Muhammad Ismail Khan[3],

[1]Department of Medical Biochemistry, College of Medicine, Buraidah, Qassim University, KSA; [2]Department of Pathology, College of Medicine, Buraidah, Qassim University, KSA; [3]Faculty of Medicine, School of Public Health, University of Queensland, Brisbane, Australia.

[*]Corresponding author:

Dr. Zafar Rasheed, Department of Medical Biochemistry, College of Medicine, Qassim University, P.O. Box 6655, Buraidah-51452, KSA.

Email: zafarrasheed@qumed.edu.sa

Supplementary Data Fig. 1

Selection of area of cartilage slicing from the discarded OA knee tissues

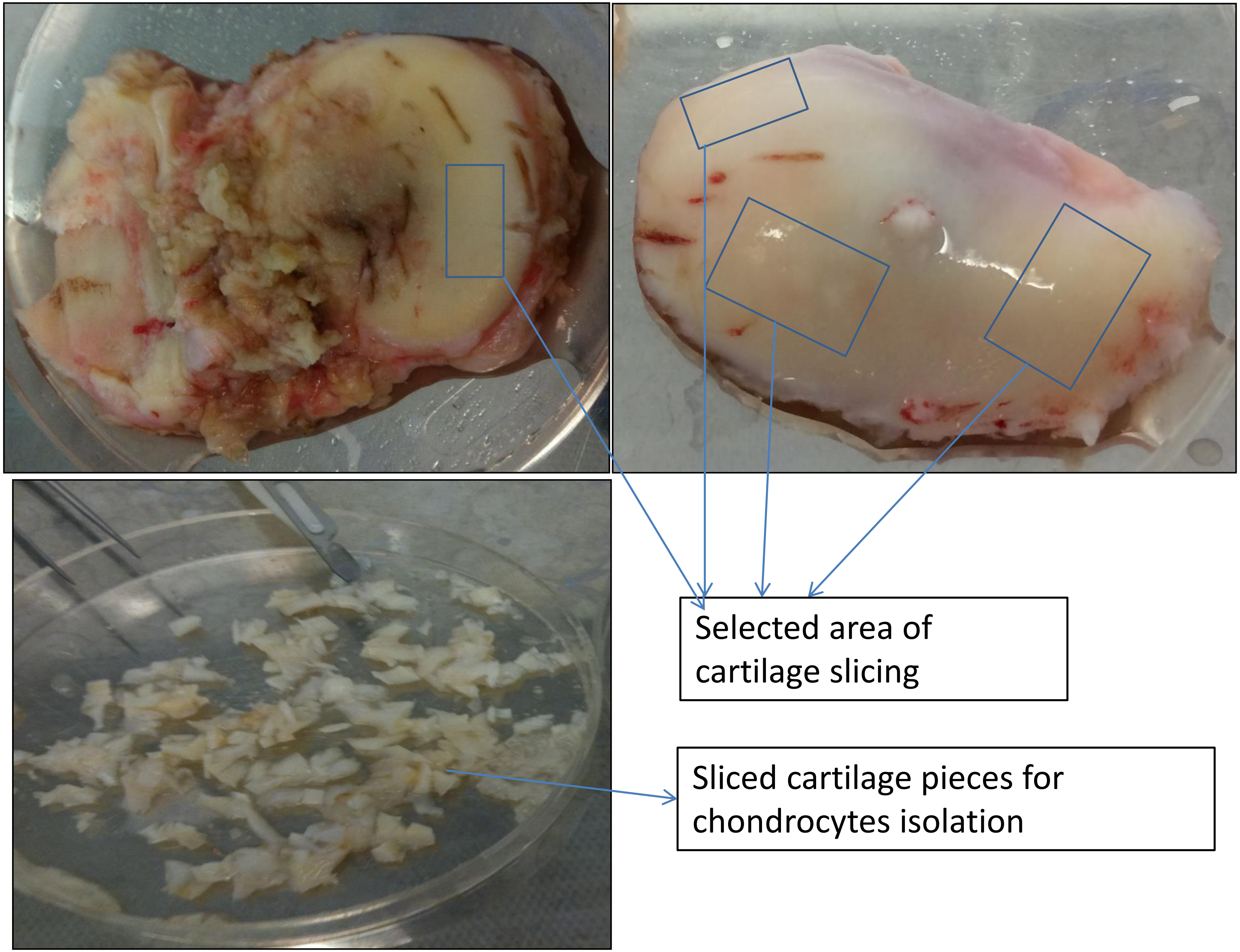

Supplementary Data Fig. 2

Figure 1 D: Lane 1 and Lane 2 were cropped

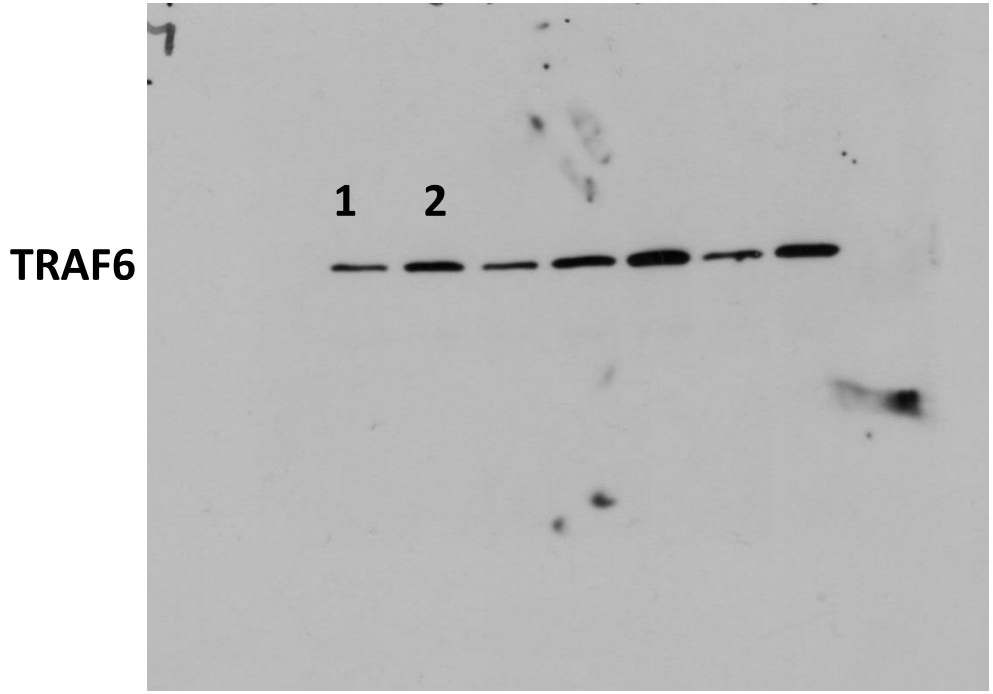

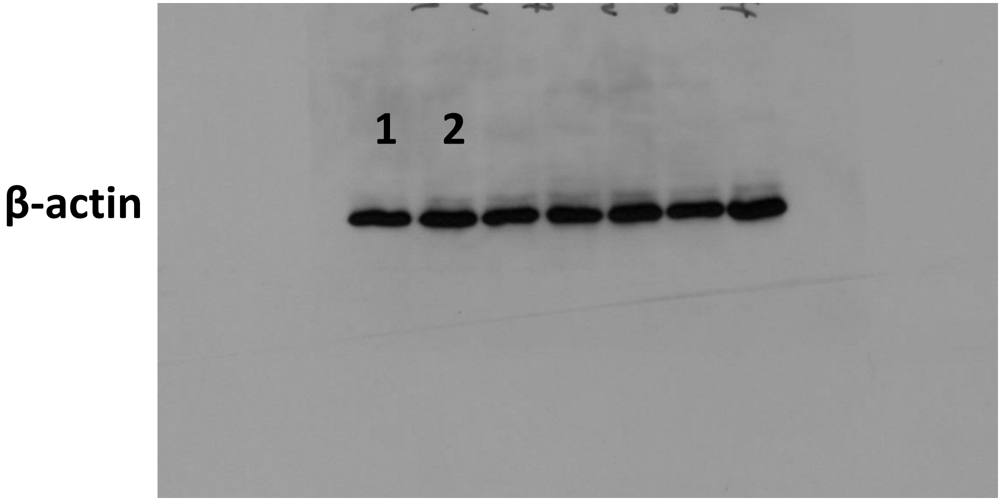

Supplementary Data Fig. 3

Figure 3 C: Lane 3 and 4 were cropped

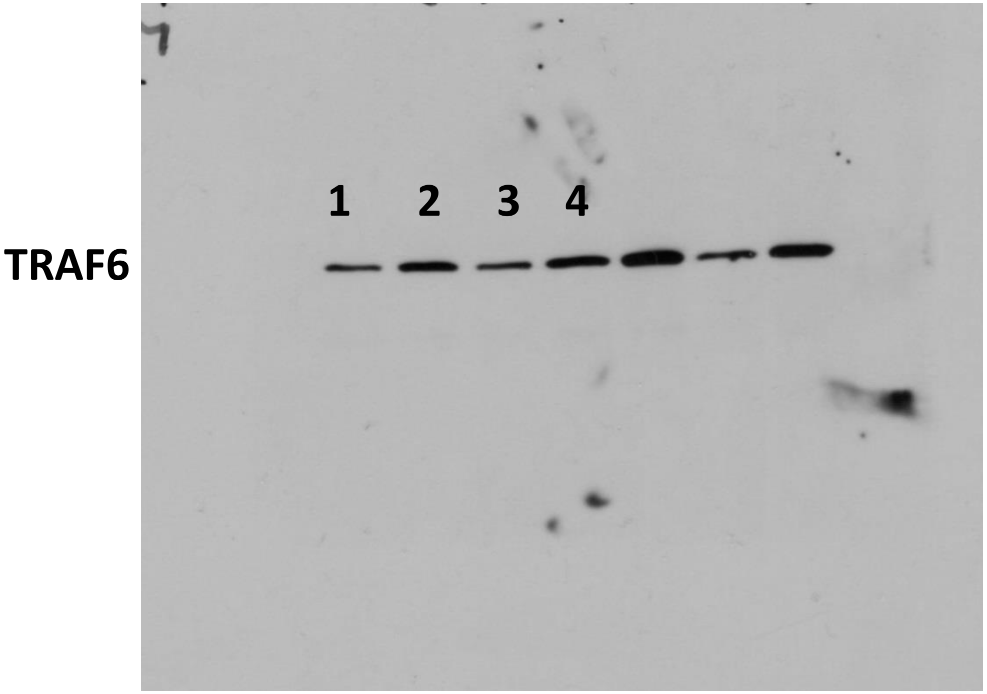

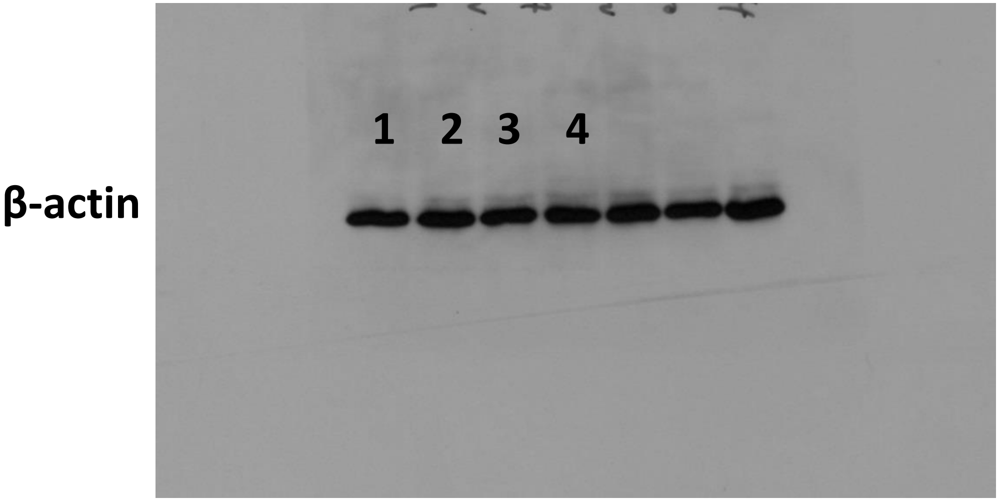

Supplementary Data Fig. 4

Figure 3 F: Lane 1, 2, 3, 4, 5 and 6 were cropped

TRAF6

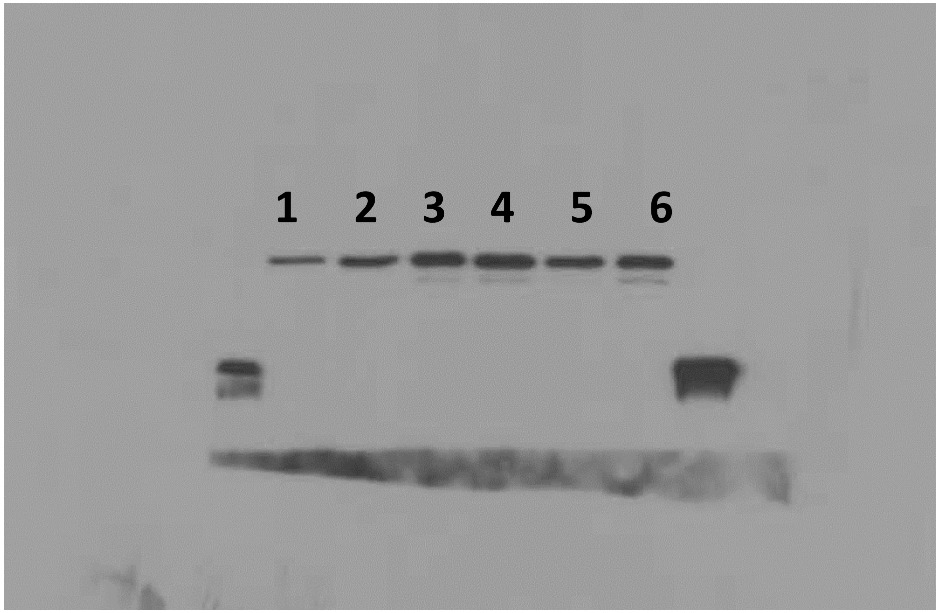

β-actin

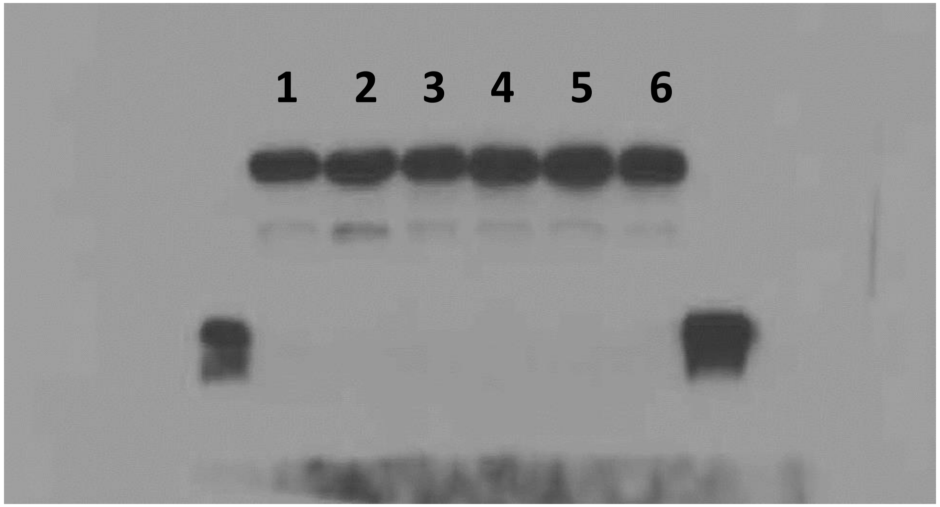

Supplementary Data Fig. 5

Figure 4 A: Lane 5, 6, 7, 8, 9, and 10 were cropped

P-p38-MAPK

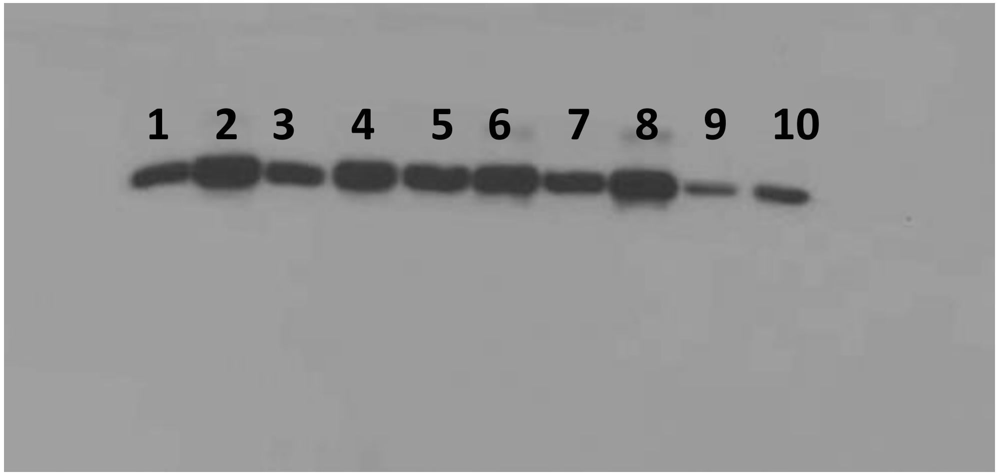

P-38-MAPK

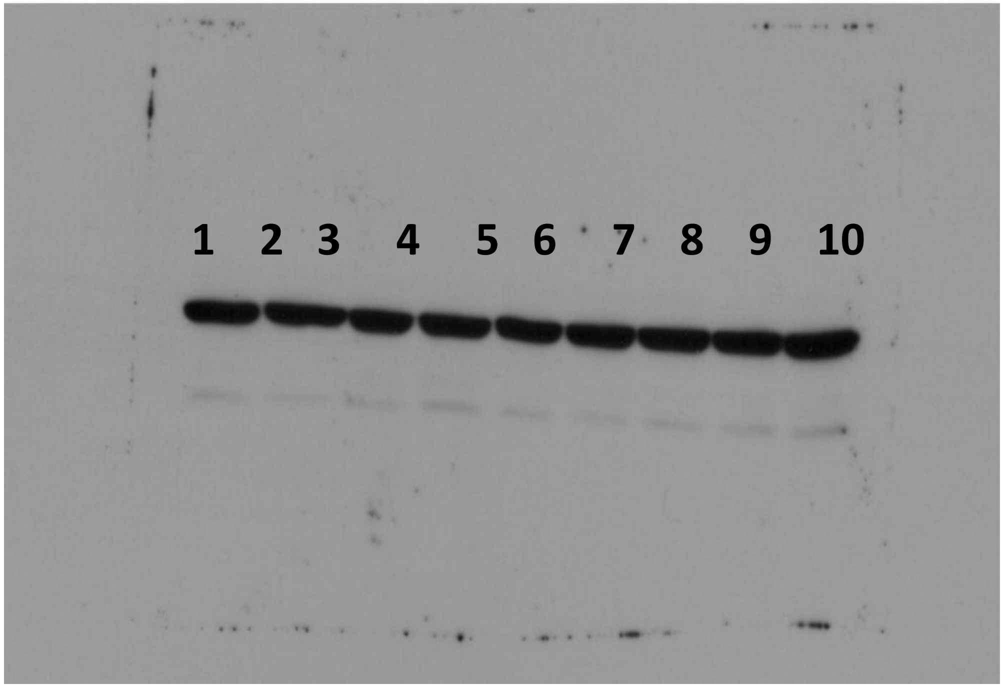

Supplementary Data Fig. 6

Figure 4 C: Lane 1, 2, 3, 4, 5, and 6 were cropped

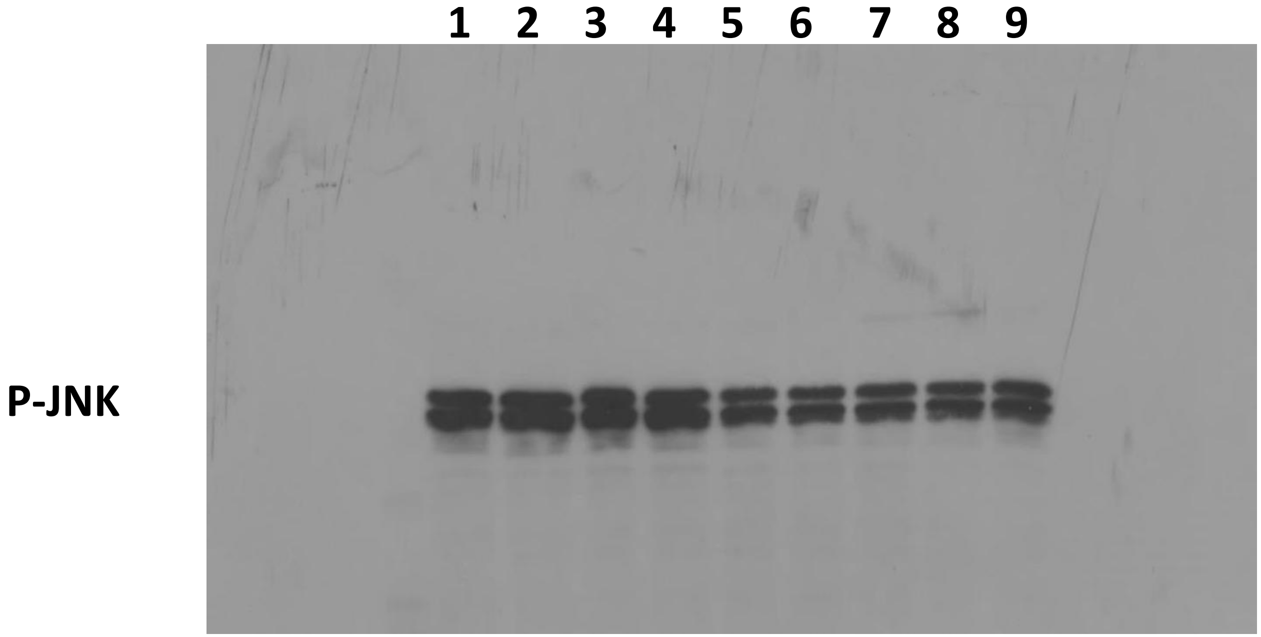

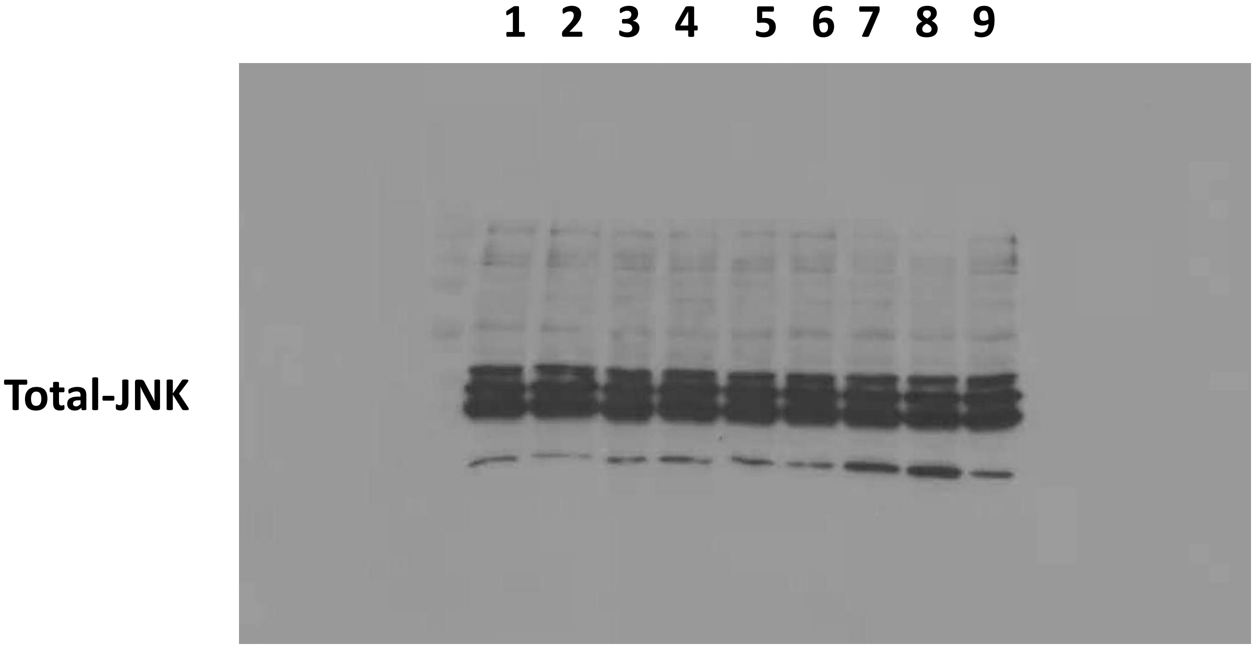

Supplementary Data Fig. 7

Figure 4 E: Lane 1, 2, 3, 4, 5 and 6 were cropped

1 2 3 4 5 6 7 8 9

P-ERK1/2

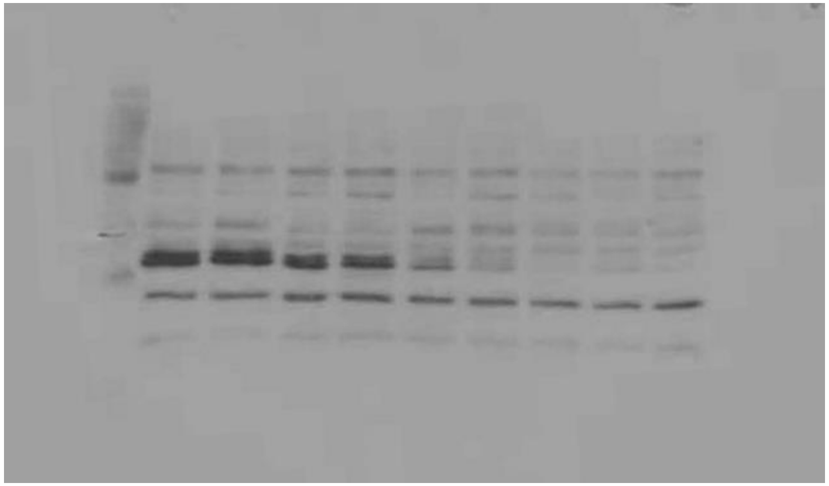

1 2 3 4 5 6 7 8 9

Total-ERK1/2

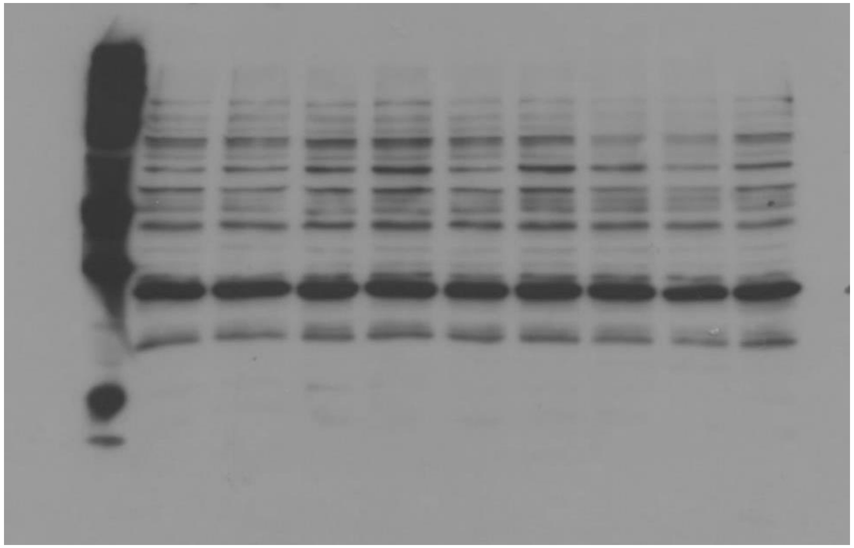

Supplementary Data Fig. 8

Figure 5 C: Lane 1, 2, 3, 4, 5, and 6 were cropped

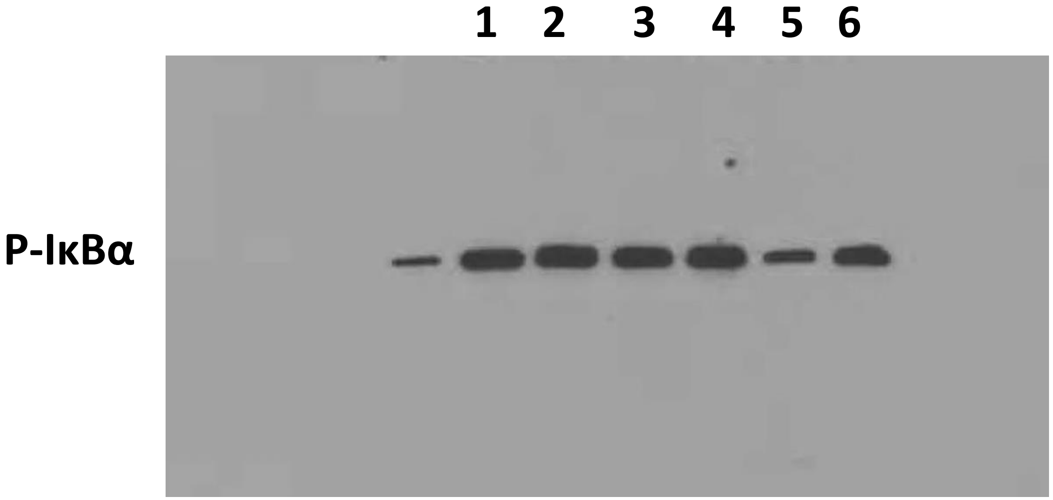

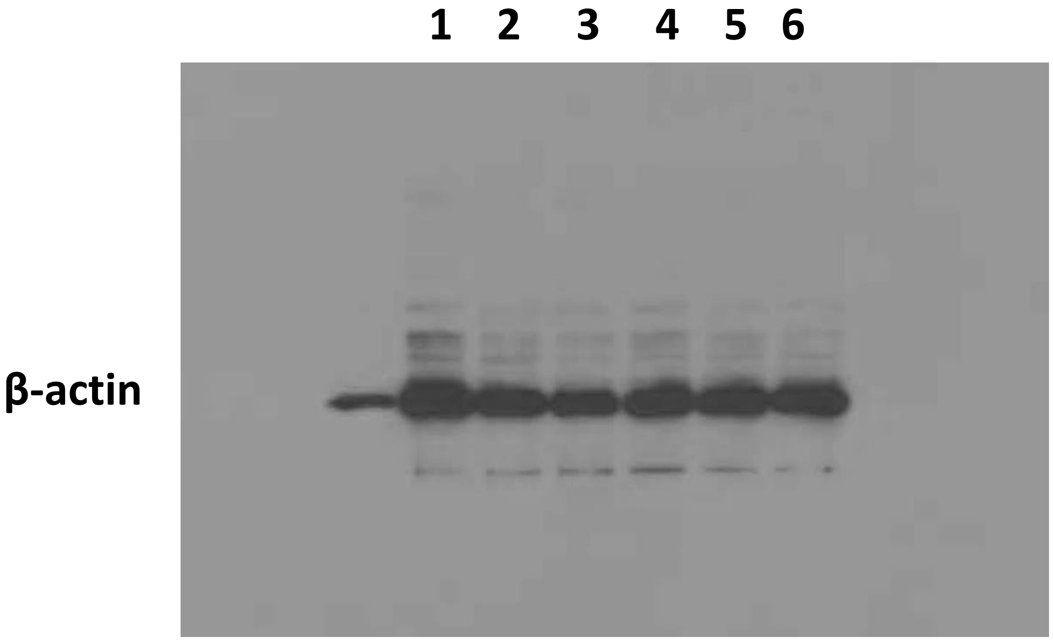

Supplementary Data Fig. 9

Figure 5 F: Lane 1, 2 and 3 were cropped

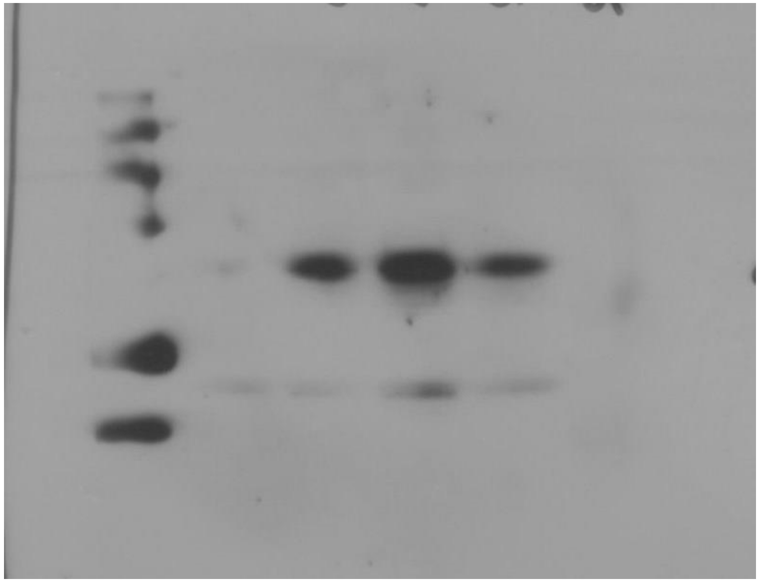

Supplementary Data Fig. 10

Figure 6 B: Lane 1, 2, 3, 4, 5, 6, 7 and 8 were cropped

1 2 3 4 5 6 7 8

MMP-13

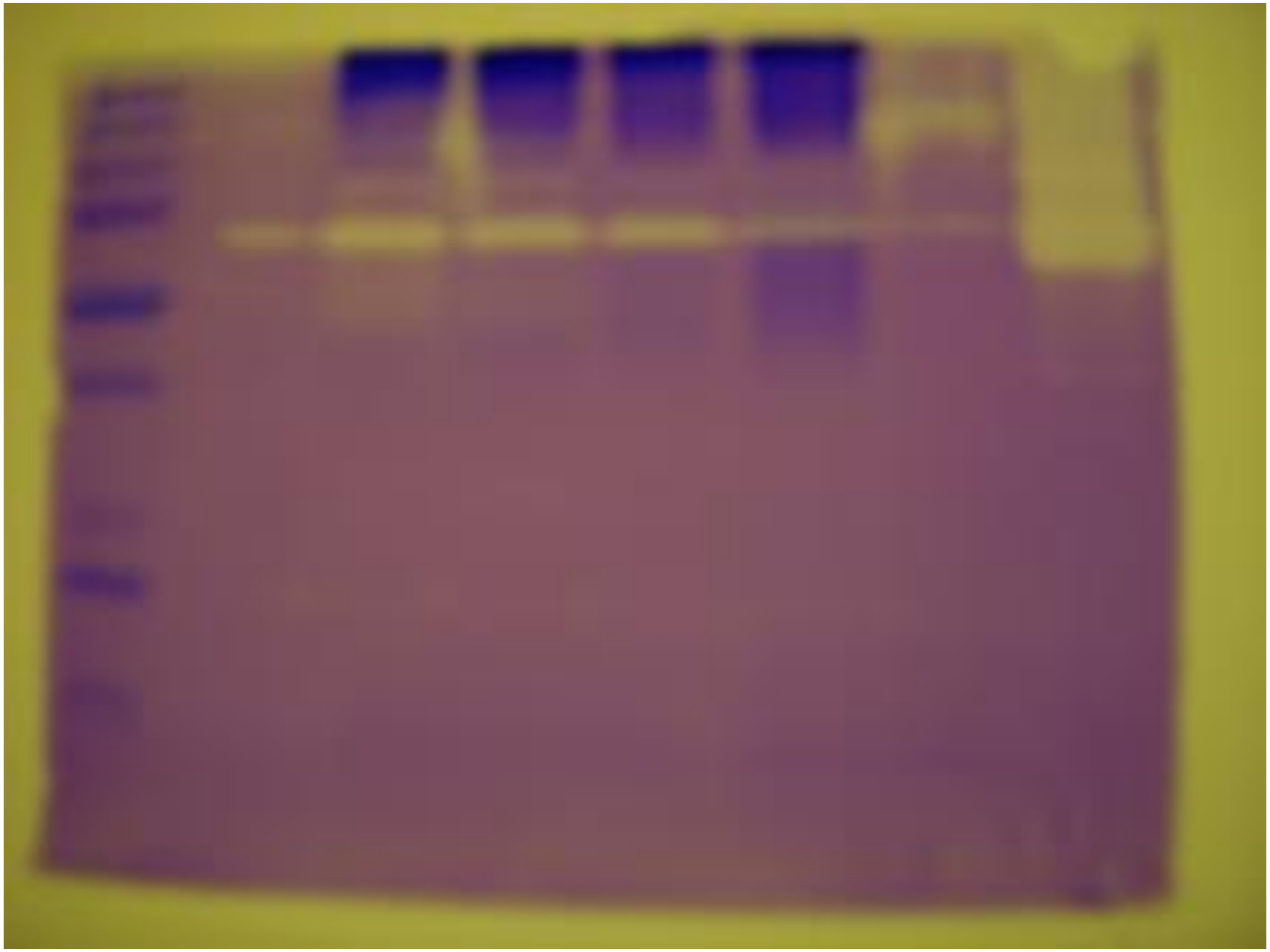

Supplement: Supplementary file 1 — Supplementary Data [file 41598_2019_42601_MOESM1_ESM.pdf]
